# Suggestions as to the Means of Procuring an Efficient Law for the Registration of Births, Marriages, and Deaths, in the State of New York

**Published:** 1853-01

**Authors:** 


ART. III. — *Suggestions as to the means of procuring an efficient law for the Registration of Births, Marriages, and Deaths, in the State of New York.*

The present law, all will agree, is good for nothing. It is equally true that were its provisions to be fully carried out, it would furnish a most valuable source of vital statistics. This point I do not intend to discuss as all medical men are agreed upon it.

What then are the reasons that a law in itself desirable does not work?

The writer has for a year past held the office of town superintendent of Schools, and the details of his duties in that function have suggested to him a plan by which he believes the law can be carried out. The present provision is this: The school district clerk reports to the town clerk the number of births, marriages, and deaths; the town clerk reports to the county clerk, and the county clerk to the Department of State.

School district clerks are, upon a liberal average, a thick-headed race of mortals, having no interest in the law. The same may be said of town clerks.

District clerks have no other duty similar to this, and moreover have no official intercourse with the town clerk, save in this instance. In many cases it requires a special journey of six or eight miles, for the district clerk to make his report. It is, in every way, extremely inconvenient, and out of his accustomed line of action, for the district clerk to perform this duty; and the result is, it is left unperformed.

A parity of circumstance applies to the town clerk. He, at the season of the year in which his report is required, has no other official business with the county clerk, and caring nothing about the law, and receiving few or none of the district report, he fails to perform his duties.

Whether any penalties are attached to nonfulfillment I do not know. If there are any they are never exacted.

Now for my plan: A law of which self-interest guarantees the fulfillment, requires the *Trustees* of school districts annually to report the number of children in their district between the ages of four and twenty-one. This law is unfailingly complied with, as on it depends the school money of the district. Now, oblige trustees to *include in this report* the number of births, marriages, and deaths. Have the town superintendent, to whom the report is made, and who is generally a man of some education, and likely to feel interested in the law, withhold from all districts failing to report, their quota of public money, and finally let the blank reports which are annually furnished to trustees contain the necessary forms.

The district reports once in the hands of the town superintendent, would unfailingly reach the State Department with his annual report made on the first of August, as self-interest again guarantees, for this report failing, no public money would be apportioned to the town. It will be seen that this plan takes the working of the law from the hands of ignorant and uninterested men, and gives it to men of education, with the strong guaranty of self-interest to insure its fulfillment. H.

